# Validating Biobehavioral Technologies for Use in Clinical Psychiatry

**DOI:** 10.3389/fpsyt.2021.503323

**Published:** 2021-06-11

**Authors:** Alex S. Cohen, Christopher R. Cox, Raymond P. Tucker, Kyle R. Mitchell, Elana K. Schwartz, Thanh P. Le, Peter W. Foltz, Terje B. Holmlund, Brita Elvevåg

**Affiliations:** ^1^Department of Psychology, Louisiana State University, Baton Rouge, LA, United States; ^2^Center for Computation and Technology Louisiana State University, Baton Rouge, LA, United States; ^3^Department of Psychology, University of Colorado, Boulder, CO, United States; ^4^Department of Clinical Medicine, University of Tromsø—The Arctic University of Norway, Tromsø, Norway; ^5^The Norwegian Center for eHealth Research, University Hospital of North Norway, Tromsø, Norway

**Keywords:** digital phenotyping, serious mental illness, clinical science, psychiatric illness, biobehavioral, psychometrics

## Abstract

The last decade has witnessed the development of sophisticated biobehavioral and genetic, ambulatory, and other measures that promise unprecedented insight into psychiatric disorders. As yet, clinical sciences have struggled with implementing these objective measures and they have yet to move beyond “proof of concept.” In part, this struggle reflects a traditional, and conceptually flawed, application of traditional psychometrics (i.e., reliability and validity) for evaluating them. This paper focuses on “resolution,” concerning the degree to which changes in a signal can be detected and quantified, which is central to measurement evaluation in informatics, engineering, computational and biomedical sciences. We define and discuss resolution in terms of traditional reliability and validity evaluation for psychiatric measures, then highlight its importance in a study using acoustic features to predict self-injurious thoughts/behaviors (SITB). This study involved tracking natural language and self-reported symptoms in 124 psychiatric patients: (a) over 5–14 recording sessions, collected using a smart phone application, and (b) during a clinical interview. Importantly, the scope of these measures varied as a function of time (minutes, weeks) and spatial setting (i.e., smart phone vs. interview). Regarding reliability, acoustic features were temporally unstable until we specified the level of temporal/spatial resolution. Regarding validity, accuracy based on machine learning of acoustic features predicting SITB varied as a function of resolution. High accuracy was achieved (i.e., ~87%), but only when the acoustic and SITB measures were “temporally-matched” in resolution was the model generalizable to new data. Unlocking the potential of biobehavioral technologies for clinical psychiatry will require careful consideration of resolution.

## Introduction

The 1850's cholera epidemic in the SOHO district of the United Kingdom serves as a stark example of the need for precision measures in medicine (1). In response to a rising death toll, experts tracked and measured mortality associated with the disease; a metric with near perfect consistency across experts (i.e., inter-rater reliability) and time (i.e., test-retest reliability) and a high degree of face, convergent, predictive and construct validity (i.e., death). Yet this measure was insufficient for accurately understanding the problem and led to ineffective causal theories (e.g., the “miasma” theory—that toxic air was causing the illness) and treatments. It was not until Dr. John Snow began mapping positive and negative cases in three-dimensional space that the epicenter of the health crisis was identified and resolved (i.e., the responsible water pump was disabled), its bacterial cause was identified, and a treatment/cure could be realized. This landmark event illustrates how traditional psychometrics, focusing on gross levels of reliability and validity, can be insufficient for developing tools that successfully understand, treat and cure pathology.

A similar situation is occurring in clinical sciences. Clinical psychiatric/psychological disorders reflect one of the most economically costly and deleterious conditions known to humankind (2). Despite thousands of reliable and valid clinical measures in use, our understanding of these disorders is generally poor and existing treatments are palliative instead of curative and preventative. The 21st century is witness to the development of highly sophisticated measures with increased attention to biobehavioral and genetic measures, ambulatory assessments, and other measures that promise unprecedented insight into psychiatric disorders (2–6). This involves, at least in part, the development and application of relatively inexpensive “biobehavioral” measures that evaluate specific channels of objectively-defined behavior tied to key neurobiological functions, for example, through the use of portable electroencephalography, eye-tracking and facial and speech analysis. Many of these technologies yield continuous data streams that can be collected unobtrusively while a patient navigates their daily routine—thus extending assessment well-beyond the confines of the traditional clinical setting. Complementing these methods are novel models of serious mental illness that focus less on subjective and clinically observable phenomena and more on psychopathology across levels of complexity within the central nervous system [e.g., the Research Domain Criteria initiative; (7)]. Collectively, these advances promise to provide low-cost and time-efficient procedures translatable to a wide array of clinical and non-clinical settings, and in doing so, can yield unprecedented objective, large-scale data sets on the nature of psychiatric diseases. This in turn can inform interventions by facilitating biofeedback, optimizing pharmacological type/dosing, improving psychosocial intervention efficiency, and personalizing interventions more generally. However, behavioral and neuro-sciences have struggled with evaluating and implementing objective measures capable of effectively predicting, diagnosing, or treating psychopathology (8–10). It is the thesis of this paper that the application of clinical psychometrics will need to change, borrowing from bioinformatics, engineering, computational and other sciences. Seeing past the “psychopathology miasma” that currently hampers scientific discovery of psychological/psychiatric disease may require a focus on “resolution.”

## Classical Test Theory and Resolution

Despite statistical and methodological improvements in the field of psychometrics since its birth in the 19th century, their application to measures of psychiatric disease have remained relatively consistent. This involves a focus on reliability and validity. Reliability concerns the consistency of a measure: across individual items of a measure (e.g., internal consistency), time (test-retest reliability), informants (e.g., inter-rater reliability), and situations (e.g., situational reliability). Validity concerns the accuracy of the measure and is evaluated based on convergence with clinically-relevant criterion (concurrent and predictive criterion validity), relationships with conceptually related (e.g., convergent measure) and unrelated (e.g., discriminant validity) constructs, conceptual comprehensiveness (e.g., content validity), and putative structure (e.g., structural validity). Importantly, the reliability and validity of measures in clinical psychology and psychiatry is far below what would be acceptable in other sciences. For example, test-retest and inter-rater reliability values explaining 50–70% of score variance are generally considered moderate and good, respectively (i.e., Intra-Class Correlation Coefficients > 0.50 & 0.70) (11). Values for the most important and popular psychiatric clinical measures, such as the Structured Clinical Interview for the DSM IV (e.g., interrater reliability = 0.47–0.80) (12), cognitive measures (e.g., test-retest of the MATRICS Consensus Cognitive Battery = 0.60–0.84) (13), symptom measures and self-report scales (e.g., test-retest and internal consistency of Beck Depression Inventory = 0.69 and 0.84) (14) are generally in this range. In physics, chemistry, engineering, biological, computer, informatics and other sciences concerned with measurement error, such high levels of unexplained variance and uncertainty would generally be considered unacceptable and associated with potentially catastrophic outcomes. In these fields, “resolution” is a critical factor in evaluating and optimizing the reliability and validity of a measure.

Human behavior is complex—and many factors contributing to this complexity likely constrain reliability and validity estimates. “Resolution” offers the ability to systematically quantify these factors. Resolution concerns the degree to which changes in a signal can be detected and quantified. Practically speaking, resolution helps optimize information for specific inferential purposes. There are three types of resolution potentially relevant to clinical measures: temporal, spatial, and spectral. Temporal resolution concerns the ability to discern information conveyed across time and is measured in units of time (e.g., seconds, minutes, weeks, years). Spatial resolution concerns how information is conveyed across physical (e.g., pixels, voxels, feet, miles, seasons), virtual (e.g., degrees of familiarity in a social network), and semantic spaces within a single time frame. It includes the ability to discern relationships of an “object” with other “objects” within this space. Spectral resolution concerns the ability to discern various aspects and subcomponents of a phenomenon. A physical example of spectral resolution involves the manner in which a prism decomposes light into its various wavelengths across the spectrum visible to humans. Definitions and examples are provided in Table 1.

**Table 1:** “Resolution” types discussed in this article.

	**Temporal**	**Spatial**	**Spectral**
General focus	The ability to discern information conveyed across time.	The ability to discern information conveyed across physical, virtual and semantic space.	The ability to discern information conveyed across subcomponents.
Physical examples of measurement units	Seconds, annual seasons	Meters, pixels	Electromagnetic wavelengths
Application to self-injurious thoughts/behaviors	The ability to discern SITB variability as a function of daily circadian and seasonal patterns.	The ability to discern SITB variability as a function of proximity to trauma-related cues.	The ability to discern intensities of various levels of SITB and its subcomponents.

**Figure 1:**
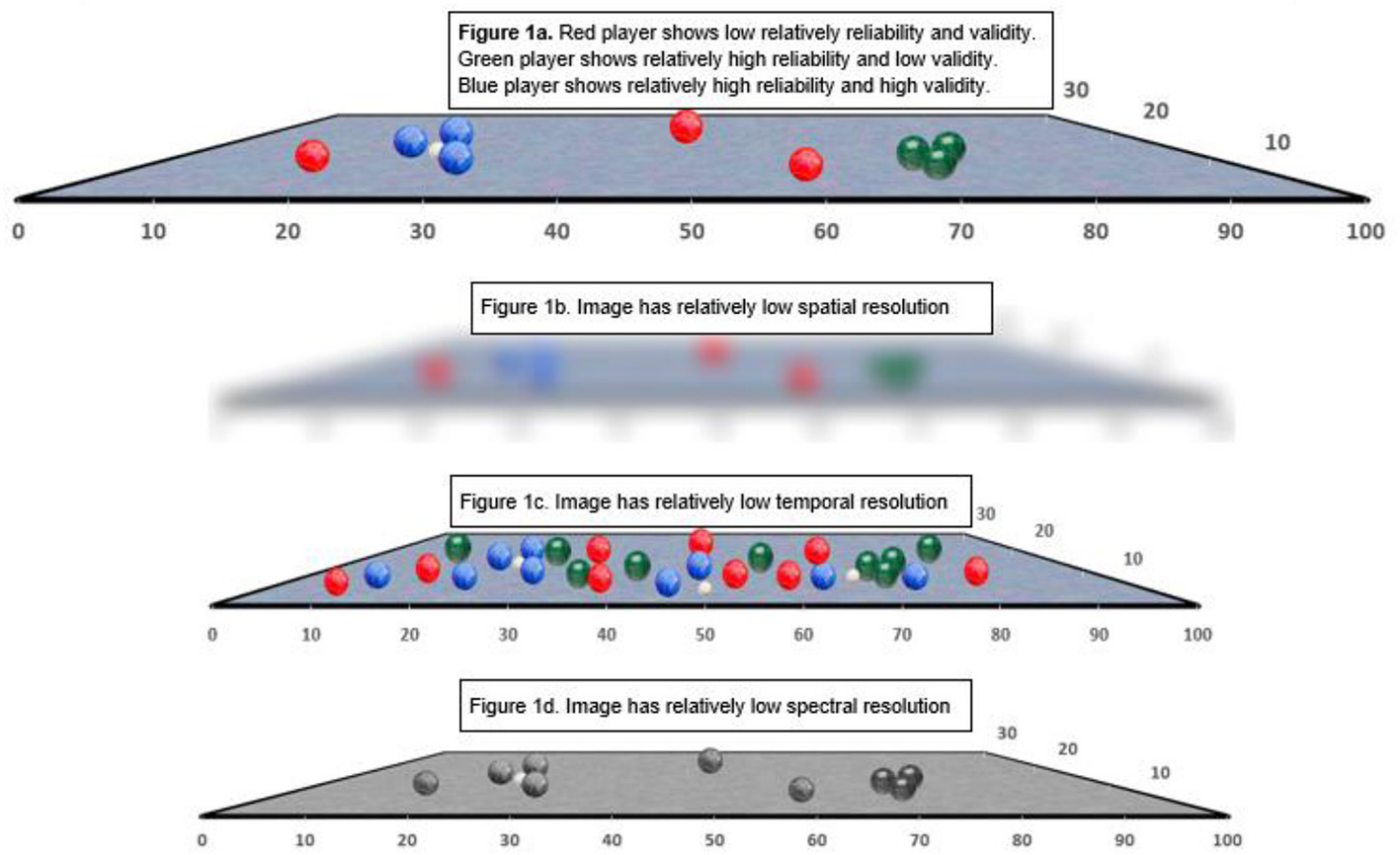
Bocci ball analogy to demonstrate reliability, validity, and the importance of resolution. **(A)** Red player shows relatively low reliability, and validity. Green player shows relatively high reliability and low validity. Blue player shows relatively high reliability and high validity. **(B)** Image has relatively low spatial resolution. **(C)** Image has relatively low temporal resolution. **(D)** Image has relatively low spectral resolution.

To illustrate how resolution can affect informatics and ultimately constrain reliability and validity estimates, a bocce ball analogy is presented in Figure 1. This analogy follows the “dartboard” analogy typically used to illustrate reliability and validity. The object of the game is for players to throw their colored bocce balls as close to the target (the “jack,” a white colored ball) as possible. Following completion of a “frame” (when all the teams' balls have been thrown), points are awarded to a single team based on the number of balls closest to the jack. Gameplay involves multiple frames, until a team reaches a predetermined score. To effectively play the game, one must be able to discern the various teams' bocce balls from each other (spectral resolution), discern the relative distances from the bocce balls to the jack (spatial resolution), and discern the various frames from each other for scoring purposes (temporal resolution). Court 1a shows how reliability and validity is typically expressed; in this case, between teams using red, green and blue bocce balls during a single frame. This information is critical for establishing the winner of each frame. Courts 1b through 1d highlight how this process is affected by resolution. In Court 1b, there is insufficient spatial resolution, hence obscuring the relative location of the balls and the jack in dimensional space. In contrast, Court 1c suffers from insufficient temporal resolution, obscuring the balls from various frames played throughout the game. Court 1d suffers from insufficient spectral resolution, making it difficult to discern information regarding the “subcomponent” teams from each other.

Within traditional clinical psychometrics, increasing levels of reliability and validity are almost invariably interpreted as desirable. This is not the case with resolution, which can only be interpreted with respect to the measure's intent. If resolution is too low, as in the case of Figure 1B, there is insufficient information for measuring one signal of interest, namely the exact distances between the bocce balls and the jack. If spatial resolution is high, but the measurements are collected over an unsuitably long timeframe, as in the case of Figure 1C, there is too much information and one cannot effectively separate signal of interest from “noise” of irrelevant bocce balls aimed at an unidentified jack. However, if we are primarily interested in whether teams have taken similar numbers of turns, Figure 1B is best because the low spatial resolution allows for highly efficient visual feature detection of red, blue and green colors on the court. On the other hand, Figure 1C would be optimal for understanding whether any player had successfully hit the jack that day, or whether there are imperfections in the court that affect game play. Importantly, many contemporary measures, particularly those that encode data digitally, are not static with respect to resolution. Rather, they facilitate dynamic resolution. Hence, spatial, temporal, and spectral resolution are scalable based on user-defined parameters. Consider on-line digital maps that provide interfaces for modifying the spatial (e.g., area scale), spectral (e.g., including geographic, roadway, business, traffic, weather, and other information) and even temporal information. Digital map use has been greatly enhanced by graphical interfaces that allow the user to view results in real time while manipulating their resolution, effectively optimizing information to answer specific questions. In the next sections, we explore how psychiatric phenotypes, such as reflected in self-injurious thoughts and behaviors, hostility and psychosis, are scalable across time, space and subcomponent and why this is critical for psychometric evaluation.

## Resolution and Clinical Psychometrics

Most psychiatric, psychological, and related clinical constructs are scalable, dynamic across time and space, and are multidimensional in structure. Note that our use of “space” here refers to proximity of a variety of factors potentially affecting the construct during a single timeframe, including those in physical, virtual and even conceptual/semantic “spaces.” Using ecological momentary analysis, for example, it is well-known that positive emotion, motivation, emotion regulation and hallucinatory experiences, vary tremendously within individuals as they navigate their daily routines, yet show predictable patterns over larger temporal epochs and when considering proximity to physical/conceptual “objects” in their spatial environment [e.g., family members, stress, arousal, fatigue; (15–18)]. Even psychological constructs considered to be “trait-like” and “immutable,” like narcissism (19), psychopathy (20), and cognition (21) appear to be dynamic over temporal and spatial epochs using high resolution measures. Understanding how psychological constructs “predictably change,” that is, show characteristic patterns across time and space, has been an emerging theme in contemporary psychopathology research. Examples include understanding “sundowning” effects in neurodegenerative disorders (22), affective dysregulation in personality disorders (23), craving in individuals with substance use disorders (17), and delusions in psychosis (18).

While clinical constructs are often scalable and dynamic with respect to resolution, data from their consequent clinical measures are often not. Structured clinical interviews, personality tests, symptom inventories, functioning measures and such generally rely on self-report information obtained cross-sectionally during a spatially- “constrained” interaction (i.e., a clinical visit). Consequently, traditional clinical measures are limited in their precision for quantifying severity of specific symptoms at a specific moment in time (e.g., for measuring social anhedonia severity between 15:00 and 17:00 last Thursday) or as a function of context (e.g., with friends vs. alone). Consider how anxiety scores from a standard clinical test compare in precision to continuous heart rate variability data. The latter data can be scaled and compared as a function of both time and context. In part, the limitations of clinical tests in this regard reflect the fact that they rarely provide ratio level data (i.e., data with an absolute zero and equal/definite ratio between data points) nor specify how these data fluctuate over defined periods of time or over clearly-operationalized spatial contexts [see (8, 23–25) for elaboration]. Consequently, they are often unable to quantify isolated channels associated with the clinical construct (i.e., poor spectral resolution), beyond general global domains associated with their factor structures.

Biobehavioral technologies offer the potential for data collection over user-defined temporal epochs (e.g., seconds, days, months), are often highly sensitive to subtle environmental changes (i.e., space), and can be constructed to simultaneously capture multiple aspects of a psychological construct. Hence, many biobehavioral technologies can be potentially scaled to measure a wide array of user-defined psychological functions. However, the dynamic nature of biobehavioral data presents a significant challenge in terms of their psychometric evaluation and their consequent analysis. High-temporal resolution measures collected in variable contexts are capturing sources of variance not necessarily present in traditional measures, and may therefore be unstable over short temporal epochs. Moreover, to the extent that the latter are used to validate the former, lack of convergence between them could be interpreted as a failure of the high-resolution biobehavioral measures to carry useful information. This is not necessarily true: as they are capturing different information and may have additional layers of complexity to account for. These concerns motivate a validation approach that considers the temporal, spatial and spectral resolution not only of the biobehavioral measure under development, but also of the criterion measures being used for validation. To illustrate this point, we examine natural vocal acoustic data from a large corpus of psychiatric patients in their variability over time and “space” (i.e., spatial conditions/resolutions) and in their ability to predict clinical phenomenon, namely self-injurious thoughts and behaviors (SITB).

## Illustrating the Importance of Resolution: Modeling Self-Injurious Thoughts/Behaviors With Acoustic Vocal Features

### Background

Acoustic vocal analysis involves quantifying aspects of vocal expression such as pitch, intonation, emphasis, vocal rate and speech production. Acoustic vocal analysis can be automated, is generally inexpensive, and uses behavioral samples that can be collected using many different modalities/media (e.g., using telephone and ambient recording procedures). Vocal expression is informative for understanding a wide range of emotional, cognitive and psychiatric states [e.g., (26–28)], and is often abnormal in the presence of SITB [e.g., see (29) for a recent review of this literature]. Hence, acoustic analysis has been proposed as an efficient, objective and potentially automated measure of SITB translatable to clinical settings (e.g., crisis phone centers, emergency rooms). To date, acoustic measures of SITB has been the focus of over a dozen peer-reviewed studies and scientific proceedings [e.g., (30–39)] and legal patents.

Despite promising “proof of concept” research, acoustic technologies have yet to be implemented in any clinical settings or approved by any governmental regulatory agency for prediction of SITB. In part, this reflects a general lack of replication of any specific methods and findings across studies. Early studies suggested that specific acoustic features, notably related to “jitter,” were particularly useful for identifying SITB (33, 35). However, subsequent research has failed to uniformly replicate this, and has focused on much broader acoustic feature sets, including those encompassing fundamental frequency, intensity, formant frequency, spectral analysis, articulation rate and pause behaviors. These feature sets range from singular to large (i.e., over 6,000 features), procured using a variety of speaking tasks, and analyzed using a variety of hypothesis driven and exploratory procedures. Moreover, measures of SITB vary widely by study, for example, using clinical rating scales (e.g., Hamilton Depression Rating Scales, as in Hashim et al. (34); Columbia Suicide Severity Rating Scale, as in Venek et al. (38), and/or recent suicide attempt behavior [e.g., (33)]).

Resolution is critical for both interpreting the extant literature and for extending it. Although prior studies of SITB have generally not examined temporal stability, acoustic features show considerable variability as a function of an individual's emotional state, context, speaking task and other “spatial” and “temporal” factors (26, 40–42). Hence, reliability should be low to modest unless accounting for these factors, as they would be expected to naturally fluctuate. Kelso et al. (41) provided a conceptual framework for understanding how relatively low-level features of speech articulation are highly dynamic, and difficult if not impossible to interpret without contextual information. Building on this, we have demonstrated how spatial resolution of acoustic features affects stability of natural speech recorded from smart phones over a 1-week epoch in patients with schizophrenia and non-psychiatric controls (24). When not accounting for contextual factors (e.g., “what type of activity are you engaged in prior to speaking?), the consistency of acoustic features was poor [i.e., <0.50; (43)], with a range of Intra-class Correlation Coefficient values [ICC] 0.00–0.48. When accounting for obvious contextual factors (e.g., in a social setting, at home/work), ICC values increased appreciably, with many exceeding moderate and acceptable thresholds [i.e., 0.50 and 0.75, respectively; (43)].

Resolution is also important for understanding concurrent and predictive validity, as validity should be constrained when acoustic features and SITB measures are grossly unmatched in temporal and spatial resolution. Typically, “gold standard” SITB measures (e.g., HAMD, CSSR) are used for validation; measures that cover a broad, variable or ambiguous swath of time and are often imprecise with respect to setting [e.g., cover a broad range of settings; (44)]. This is problematic in light of increasing evidence that SITB is highly variable across brief temporal epochs (45), and more importantly, that the acoustic sampling was likely conducted hours, days, or weeks from the SITB assessment and in an entirely different context. It is true that machine learning based algorithms have demonstrated impressive accuracy for predicting “gold standard” SITB measures [e.g., (36, 46)] with accuracy far exceeding the near-chance levels seen with clinical judgement (47). However, to our knowledge, generalizability to other “resolutions” settings, speaking tasks and measures of suicidality have not been demonstrated [a point alluded to in Ribeiro et al. (48)]. In support of this generalizability concern, Walsh et al. (46) employed machine learning of clinical history and demographic information to predict SITB at various time points (e.g., 1-week, 1-month, 1-year). While their models showed impressive accuracy, the model features and weights varied considerably across these time points (46). It is our thesis that optimizing the reliability, validity and generalizability of acoustic analysis for measuring SITB requires consideration of temporal and spatial resolution.

In the following section, we examine a limited vocal feature set in predicting SITB in a sample of inpatient and outpatients with various psychiatric diagnoses. These analyses are constrained by the nature of our data and were meant to explore the role of resolution on reliability and validity using “real-world” data, as opposed to developing generalizable algorithms for SITB prediction. SITB was examined across two different temporal resolutions: momentary [i.e., ratings based on Ecological Momentary Assessment (EMA) within 5-min of vocalization], and 2-week (i.e., clinical ratings from the prior 2-weeks) epochs. Vocal samples were evaluated across ambulatory (i.e., recorded from a mobile device) and clinical interview (i.e., recorded during a structured clinical interview) assessment formats. While perhaps a bit esoteric, this recording format reflects a kind of spatial resolution in their systematic differences in terms of where they lie in a semantically-defined “assessment space.” The clinical interview format reflects a traditional assessment domain characterized by a semi-structured dyadic interaction whereas the ambulatory format reflects a more novel assessment domain using pre-recorded, technology-based interaction. We examined how temporal reliability and concurrent validity of these vocal features change as a function of these temporal and spatial resolutions, and how they change when the vocal data are scaled to match the criterion measure (i.e., from seconds to weeks). It was our expectation that models with acceptable reliability and validity could be established, but only when examined as a function of the aforementioned temporal/spatial resolutions. We did not examine SITB as a function of spectral resolution for lack of data, though it is widely regarded that SITB is comprised of at least several subcomponents (49).

## Methods and Materials

### Participants

Data presented here were part of several studies that employed a mobile application for longitudinally tracking mental states of psychiatric patients (50–57). The software application—*delta* Mental Status Examination (*d*MSE), comprises a number of assessment tasks that engage participants in spoken and touch-based interactions in order to capture daily measures of cognition, affect, and clinical state for tracking various SMI-related risk states. Patients were 25 stable outpatients with SMI (i.e., actively being treated for a schizophrenia, mania or depression-spectrum disorder) recruited from a community group home and 99 psychiatric inpatients recruited from a community-based substance-use treatment facility. Approximately a third of the sample met criteria for major depressive disorder lifetime (*n* = 40), 15 percent met criteria for schizophrenia (*n* = 19), and a minority met criteria for bipolar disorder (*n* = 7). The remainder of patients suffered from various substance use, personality, anxiety and other depression-spectrum disorders (not formally assessed in this study). The sample was predominantly male (96%), in large part, because the inpatient facility exclusively admitted men. The sample was African-American (56%) and Caucasian (44%). Most of the participants had earned a high school or equivalent degree (70%), and half had at least some college or university education (50%). The average age of the participants was 38.54 years (standard deviation = 11.05). Participants were free from major medical or other neurological disorders that would be expected to impair compliance with the physical production of speech or operation of a smart phone (e.g., blindness). Participants received extensive instruction on using the *d*MSE app. They were asked to find a quiet place to complete testing and were paid one dollar for completing each session. Study staff provided daily instructional and technical support as needed. Stable outpatients were asked to complete five sessions on consecutive days during business hours and inpatients were asked to complete four sessions per week during business hours for the duration of their inpatient stay (up to 28 days). Completion rates among active participants (e.g., not discharged from the inpatient facility) was excellent (i.e., > 90%). This high completion rate likely reflects the support offered by study staff and that compensation was provided directly after each administration. The Louisiana State University Institutional Review Board approved this project (IRB protocol 3618), and participants gave informed consent prior to their involvement in the study [see (56) for information about the *d*MSE and data security and protections].

### Vocal Assessments

Vocal data were examined across two different contexts, or “spatial resolutions.” The first involved an active interaction with the *d*MSE application during several standardized tasks. One involved a verbal memory recall task that was ~75 words long (range 69–85). Participants first heard a recording of the story, and then recalled the story immediately and after a delay. For the outpatient data collection this delay was on average 17 min, and for the inpatient data collection the delayed recall was collected during the next testing session (1–3 days). Participants had a maximum of 60 s to respond [see (50, 55) for more information]. Another task involved providing responses to static color images (selected to optimize vocal expression during the early validation phase of this project). Each image was displayed on the screen of the smart device and participants had up to 60 s to respond. These tasks were selected for analysis because they were both administered to the inpatient and outpatient samples, and provided sufficient speech for acoustic analysis (i.e., more than monosyllabic or two utterance responses). In the present study, these tasks were originally analyzed separately, however, they did not appreciably differ in acoustic features, so they were combined for the analyses presented here. Samples with less than three vocal utterances were excluded from this study (i.e., speech bounded by silence >150 ms in length with no contiguous pause > 50 ms). For the second spatial condition, vocal features were extracted from the video-recorded clinical interview (when available), using the first and last 5-min epochs as separate samples. The clinical interview involved a structured interview to asses DSM 5 diagnosis or symptom severity and was conducted by a trained research assistant. All interviewer speech was digitally spliced from the audio recording, and both interviewer and patient speech were removed when they were speaking simultaneously. There were 2,221 usable ambulatory and 117 interview recordings examined in this study, though the exact number analyzed varied as a function of data availability (outlined below). Approximately 39% of the ambulatory recordings were excluded because they contained less than three utterances (*K* of original ambulatory recordings = 3,656).

### Acoustic Feature Extraction

In evaluating the extant literature on acoustic analysis and SITB, it is clear that (a) a wide variety of features have demonstrated value in predicting SITB, and (b) conceptually diverse feature sets generally outperform singular ones (see intro for elaboration). Our intent in this study was not to optimize prediction of a SITB measure, but rather, to demonstrate how various features change in their reliability and validity as a function of changes in resolution. For this reason, we used a relatively simple “macroscopic” feature set defined by the Computerized Assessment of Natural Speech [CANS; (53, 58–61)]. This set, generated using various Praat scripts, was developed and validated by our research team for measuring psychiatric states. These features resemble (conceptually, if not mathematically) those with shown associations to SITB in at least some prior studies (see Table 2). Each feature has been associated with psychopathology symptoms, notably depression (29, 53, 62, 63) and most have been associated with SITB in prior research; [Fundamental Frequency (F0) mean (38, 64), F0 variability (37), jitter (33, 35), silence/pause duration/number of utterances (34, 37, 38) and formant frequencies (33, 39)].

**Table 2:** Vocal properties and features examined in this study.

**Feature**	**Function**	**Operational definition**
**“Speech variability”**		
“Pitch”	Frequency of vocal fold vibrations	Average fundamental frequency (F0; in semitones)
Intonation	Variability in F0	SD of F0 within each utterance, averaged across utterances
Emphasis	Variability in intensity/volume	SD of intensity within each utterance, averaged across utterances (in decibels)
Jitter	F0 signal perturbation	Change in F0 signal in consecutive measures, averaged across utterances
Shimmer	Intensity/volume signal perturbation	Change in intensity/volume signal in consecutive measures, averaged across utterances
**“Formant frequencies”**
F1 Variability	Vertical tongue movement	SD of F1 values within each utterance, averaged across utterances (in Hertz)
F2 Variability	Sagittal tongue movement	SD of F2 values within each utterance, averaged across utterances (in Hertz)
**“Speech production”**		
Pause mean	Pauses between vocal units	Average silence between voiced utterance (in seconds)
Number of utterances	Speech quantity	Number of voicings bounded by silence

A description of the nine vocal features used are included in Table 2. These features were selected based on prior Principal Component Analysis of 1,350 non-psychiatric adults (61), 309 patients with SMI (60) and published analysis of the 25 outpatients examined in this study (53). Importantly, the latter study did not involve any measures of SITB. The acoustic features were non-redundant, with all but three inter-correlations <0.30, and all <0.80. In terms of scaling, acoustic analysis was conducted approximately every 20 milliseconds of recording, and features are computed based on the full recording. For ambulatory testing, there were three recordings during each session, and between three and five sessions per week per participant.

### Self-Injurious Thoughts/Behavior

Two measures of SITB were used in this study. The first reflected measurement at a single moment and was procured at the same time as the vocal sample (recorded during the *d*MSE session). Hence, they were “matched” in temporal resolution. This involved a digital slider to indicate how much participants felt like “harming themselves” on a scale from one to 100. Data were available for 811 of 875 potential data points (i.e., missing for 64 samples). A lower temporal resolution measure of SITB, based on the “suicidality” item from the Brief Psychiatric Rating Scale (65) was also used; this measure of SITB was not “matched” in temporal resolution with the single moment, ambulatory vocal sample. This item involves an ordinal rating of the most severe SITB over the last 2 weeks. Information was based on self-report, medical record and staff. All clinical assessments were conducted by a licensed clinical psychologist (Alex Cohen) and his research team. Ratings and diagnoses reflect a consensus from this team. BPRS scores were collected for all 25 outpatients, and for 47 inpatients. Due to limited interviewing resources and time, we prioritized BPRS ratings for those inpatients that carried SMI diagnoses (i.e., psychosis, mania or depression-spectrum) or were actively symptomatic per medical records or staff report. BPRS data were not collected for 52 inpatients (*K* = 805 samples). For prediction purposes, the momentary (using a cut-score of 51/100 and above) and 2-week (using a cut-score of 2.1, indicating at least “mild” symptoms) measures were dichotomized. Using these criteria, 201 of 1,958 samples and 105 of 1,698 samples, respectively, met threshold for SITB. The SITB measures did not correlate highly with each other (*r* = 0.15).

### Analyses

To evaluate reliability, we computed Intra-class Correlation Coefficients (ICC) for each of nine vocal features. ICC values were based on a single measurement, two-way mixed effects model [type 1, in Koo and Li (43)], computed for all recordings, and separately as a function of two spatial resolutions (i.e., ambulatory and interview recordings), and three temporal (i.e., speaking task, or “averaged” across the testing session or averaged across 2 weeks) resolutions. We expected ICC values to be poor overall (e.g., <0.40) (11), but would increase when separately examined as a function of time and space/context, or when temporally “scaled” (i.e., averaged within the session or over a 2-week period). Given our interest in SITB, we also examined ICC in individuals with mild or greater SITB (momentary rating <51/100).

To evaluate validity, we modeled SITB as a function of our limited acoustic feature set from the speech samples using rare events logistic regression [ReLogit; (66, 67) using the Zelig package (68, 69) package for R 3.6.3 (70)]. We forced the model to behave as if SITB are observed in 50% of cases in the general population, despite constituting <5% of our sample (τ = 0.5). This amplified the penalty associated with failing to identify positive cases, thereby avoiding models that could otherwise achieve high accuracy by ignoring the acoustic features and always guess the more likely outcome (no SITB). Before modeling, the acoustic features were standardized and trimmed (values exceeding 3.5 standard deviations were replace with values of 3.5 SD).

Model accuracy was assessed with 10-fold cross-validation [cf. (71)]. The data was randomly split into ten sets, each containing a roughly equal ratio of positive and negative cases. Models were then fit to a subset of the data composed of nine sets (90% of samples) and evaluated on the one remaining set (10% of samples). This was repeated so that each set was used for evaluation. Performance metrics from each cross-validation fold were averaged before reporting. Model accuracy is computed as the average of the true positive rate and the true negative rate: (*TPR*+*TNR*)/2. This accuracy metric is both more conservative and easily interpretable than raw accuracy given how infrequent positive cases are. If the model is guessing at random (*TPR* = 0.50; *TNR* = 0.50) or is always guessing the more frequent outcome (*TPR* = 0; *TNR* = 1), accuracy is 0.50. Thus, 0.50 can always be understood to indicate that a model that is failing to discover predictive structure in the speech samples.

## Results

### Reliability

**Figure 2:**
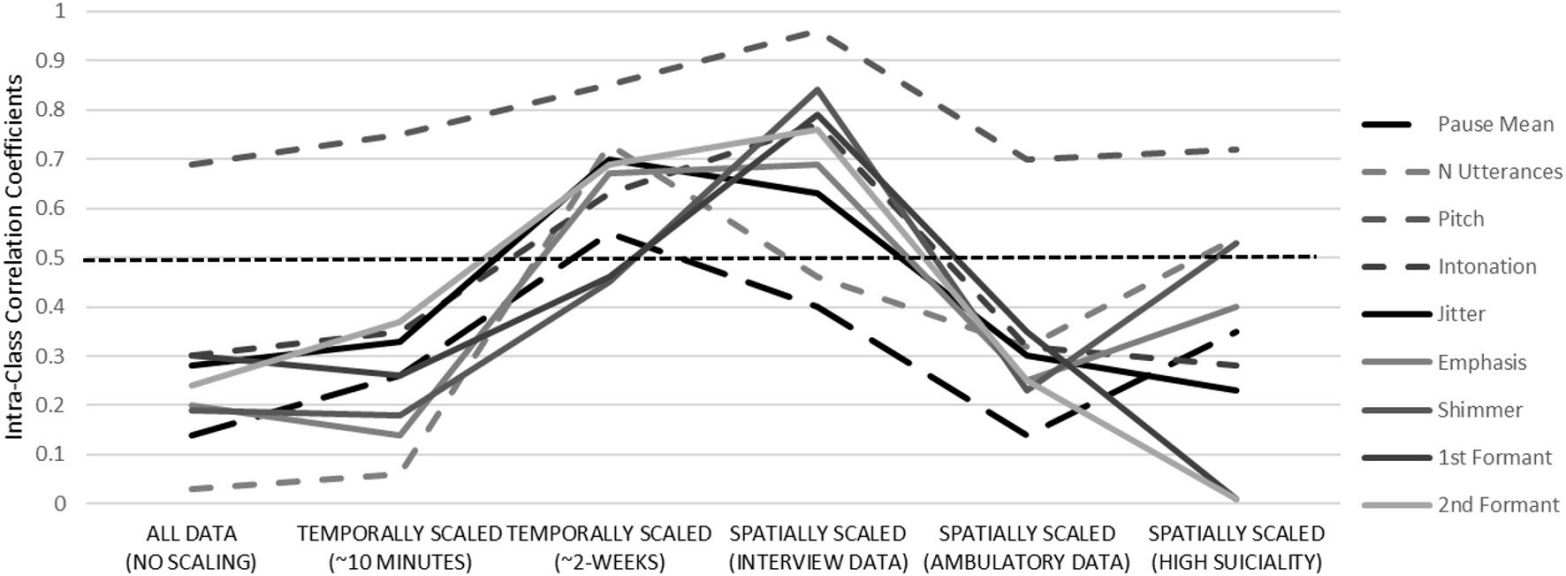
Temporal stability of acoustic features across a variety temporal and spatial resolutions. Dotted midline reflects “fair” stability, defined at 0.50. See Table 2 for definitions.

ICC values for nearly every vocal feature were low (well below 0.40) when computed without regard to temporal or spatial resolution (*K* samples = 2,335) (Figure 2). When data were “scaled” within the session (examining ambulatory data solely), ICC values did not appreciably change. When data were scaled across a 2-week epoch, moderate or greater stability was observed for each of the acoustic features (*K* = 192). With respect to spatial resolution, the stability of vocal features solely examined from the interview was impressively high, with the majority of ICC values exceeding 0.80 (*K* = 114). This was not observed for ICC values of ambulatory vocal features, which were uniformly low (*K* = 2,221). When the vocal data were scaled to include only patients reporting SITB (*K* = 85), moderate or greater stability was observed in four of nine vocal features. In sum, vocal features were unstable over time unless various contextual, setting or patient characteristics were considered. Importantly, improved stability was not solely a function of increased sampling or number of observations within individuals.

### Validity

The vocal features were modeled to fit momentary SITB, both collected with the *d*MSE application at the same time (Table 3). A model using momentary vocal features to predict momentary SITB for the training set was computed with an adjusted accuracy of 83% (*K* = 1,958). This reflected good hit (i.e., 85%) and false alarm (i.e., 19%) rates for predicting SITB. When this model was applied to the test set, an average of 83% accuracy for predicting SITB was observed across the 10-folds of the model (range = 0.72–0.91). The coefficient weights are provided in supplemental tables (see Supplementary Table 1). However, this model did not generalize well to the same SITB measure using acoustic features extracted from a different spatial resolution (i.e., the clinical interview), as 48% accuracy was observed with poor hit (22%) and false alarm (27%) rates. This model also did not generalize well to the 2-week measure of SITB (i.e., from the BPRS), where 61% adjusted accuracy was observed. This reflected relatively low rates of false positives (i.e., 24%) and true positives (i.e., 47%) for predicting SITB. Importantly, the poor generalizability from the momentary to the 2-week SITB measure did not reflect a complete disconnect between the latter and the vocal features. When a new model was computed using ambulatory vocal features to predict the 2-week measure of SITB (i.e., from the BPRS, as opposed to momentary ratings), decent accuracy was observed for the training (accuracy = 71%, true positive = 72%, false alarm = 30%) and test (accuracy across 10-folds = 742, average true positive = 77%, average false alarm = 33%) sets. This model did not meaningfully generalize to either the momentary suicidal measure or to the interview data (accuracies <56%).

**Table 3:** Modeling “momentary” and “2-week” self-injurious thoughts/behaviors based on “momentary” vocal features.

		**Time scale**	**Spatial scale**	**Model accuracy**
**Model 1: Using predictor & criterion data that are temporally matched yields good accuracy**				
Predictors:	Acoustic features	Momentary	Ambulatory recording	Adjusted accuracy: 83%
Criterion:	SITB	Momentary	Ambulatory recording	True positive: 85% False positive: 19%
**Model 2: Using predictor & criterion data that are not temporally matched yield poor accuracy**				
Predictors:	Acoustic features	Momentary	Ambulatory recording	Adjusted accuracy: 61%
Criterion:	SITB	2-Week	Clinical Interview	True positive: 47% False positive: 24%
**Model 3: Using predictor & temporally-scaled criterion data to temporally matched them improves accuracy^a^**				
Predictors:	Acoustic features	2-Week	Ambulatory recording	Adjusted accuracy: 74%
Criterion:	SITB	2-Week	Clinical interview	True positive: 67% False positive: 19%

a *Data temporally scaled by averaging data over the 2-week assessment epoch*.

To test the idea that the inaccuracy in applying these models to new data reflects a mismatch in resolution, we applied the aforementioned model using momentary acoustic features and momentary SITB (i.e., with 83% accuracy) to predict a different measure of SITB—the 2-week measure based on clinical ratings (showing 61% accuracy above). However, we temporally-scaled the former so they approximated that of the latter (i.e., including only averaged data from the 2-week epoch from which the 2-week SITB measure was derived from). This is a very preliminary test of whether matching resolution improves accuracy, as (a) there were data from 849 cases, but only 57 participants for analysis, (b) of these participants, only three had mild or higher levels of SITB, and (c) the number of sessions for extracting acoustic features varied considerable across participants, from one to 12. Nonetheless, we saw model accuracy improve to 74%. This reflected improvements over the generalization models with more true positives (i.e., 47 vs. 67%). These data are summarized in Table 3.

## Discussion

These experimental findings highlight the importance of resolution in understanding and evaluating biobehavioral data. As expected, vocal features were highly unstable over time and space. When we accounted for temporal and spatial resolution, temporal reliability improved. Replicating prior research [e.g., (29)], we were able to predict SITB with reasonable accuracy, much higher than that seen using traditional clinical measures (47, 72). The models derived using specific temporal and spatial resolution parameters did not generalize to data using different resolution parameters. However, when data from the original model were temporally scaled so they approximated that of the criterion, accuracy improved. It should be noted that, while these accuracy rates for predicting SITB far exceed chance, and those seen using clinician judgement and “gold-standard” measures (8, 47, 72), the present study was not a clinical trial. Hence, the models are insufficient for clinical implementation by themselves but are important for illustration purposes. Of note, our reliability metrics were far from the values advocated for in the beginning of this paper (e.g., 0.90 ICC values). Moreover, the models are derived from primarily male samples from one geographic region of the world using relatively constrained types of speaking tasks. There were some missing clinical data, and it is unclear how this may have impacted the findings. Finally, SITB was not well-represented in the sample and was not comprehensively measured. Nonetheless, our findings highlight the importance of resolution for future research and clinical implementation of existing models. Replication and further external validation will be key in this regard. Better understanding of potential moderating variables affecting the acoustics-SITB relationship is critical, as this knowledge can help improve generalization of the models. In lexical expression, for example, autobiographical language has been important for understanding SITB (36, 73, 74), and this knowledge could be critical for generalizing models to language from a wide array of contexts/spatial resolutions (e.g., where autobiographical reference may be more pronounced).

### How Should Resolution Be Addressed?

The importance of resolution when evaluating biobehavioral data is well-known outside of psychiatric and psychological sciences. In evaluating medical devices, for example, the US Food and Drug Administration addresses this by focusing on test-retest reliability (dubbed “precision”) and accuracy (i.e., “the degree of closeness to a known true value”) under prescribed conditions (75) the latter of which helps constrain temporal and contextual factors that influence a known signal. Evaluating accuracy as a function of “known stimuli and conditions” is common in experimental psychological research. “Manipulation checks” using unambiguous conditions and stimuli, for example, fMRI, electrophysiology and facial/vocal biobehavioral signal are often conducted using a range of physical, cognitive and emotion manipulations. Established stimuli corpuses, with standardized and well-normed stimuli, exist for this purpose [e.g., International Affective Picture System; IAPS; (76, 77)]. Examining validity when temporal and contextual factors are controlled for is also important for calibrating measures over time, in essence, reducing noise due to measurement drift.

Statistical and methodological solutions for evaluating the psychometrics of high-resolution biobehavioral measures exist. For example, “Multitrait-multimethod” matrix approach (78), and Generalizability Theory (79), provide systematic methods for differentiating potential sources of variance reflecting the construct of interest, various contextual influences, and unexplained noise. These approaches have been used, for example, to determine how ADHD ratings vary as a function of class, parent and teacher data sources (80, 81). Examples of their application to biobehavioral data include attempts to understand fMRI signal in individuals across different geographic testing sites and different testing equipment (82) and source variance of neuro-electrophysiological signal as a function of within-participant, test trial, psychiatric diagnosis and other signal factors (83). Multi-Level Modeling (MLM) approaches have also been used extensively for understanding biobehavioral data. MLM can accommodate data with a “nested” structure, such that observations are hierarchically organized within individuals, settings, and times/days. MLM, for example, has been used to understand how acoustics and facial features change as a function of time, social context and setting in schizophrenia (24, 84–86).

While current solutions can help address temporal and spatial resolution issues, a significant challenge remains in addressing the “spectral” complexity of psychiatric disorders. Compared to psychiatric diseases, cholera has a simple phenotype and pathophysiology. Hence, the solutions employed by Dr. John Snow in the 19th century may be overly simplistic for understanding psychiatric disorders. SITB, for example, can be potentially expressed across a wide spectrum of behavioral channels (e.g., facial expressions, hand gestures, language, vocal modulation), and this spectrum may vary within and between individuals over time and space. The present analyses involved a limited set of acoustic variables in this study, and some models include thousands of non-redundant acoustic features (87). When combined with the myriad of features from other behavioral domains, accurately modeling them becomes a staggeringly complex endeavor. Importantly, best practice for psychiatric assessment typically involves the use of multiple data-streams (e.g., various streams of self-report, behavioral, historical and test data) with different spatial, temporal and spectral resolution. In effect, the human brain can model these complex data and their temporal and spatial inter-relations to at least some degree. A growing field of understanding “network medicine” reflecting “networks of networks” is being developed to accommodate this complexity, though its application in clinical psychiatry has yet to be realized (88). This type of network approach could allow the end user, be they clinician, pharmacological or psychosocial treatment developer, forensic evaluator, peer, public policy analyst or the patient themselves, to reap the benefits of the new analytic approaches without being burdened with the details of the raw data or the algorithm itself.

## Summary and Conclusions

Much like cholera prior to the 19th century, psychiatric illness exacts tremendous human cost due to ignorance about its causes, and the lack of precision assessments for quantifying it. There have been repeated calls for the development of more sophisticated measures, for example, through the use of objective biobehavioral technologies, ambulatory assessment, and “big-data” analytics. Despite this, there is no obvious strategy in place for evaluating and implementing these technologies. Thus, these technologies have yet to move beyond a “proof of concept” phase. It is clear that traditional clinical psychometrics reflects an obstacle for evaluating and implementing these measures. Borrowing from computer, informatics, and physical sciences, a focus on “resolution” can help overcome this obstacle and can advance precision psychiatry in at least four ways. First, improved spatial, temporal, and spectral resolution can provide more nuanced and sensitive information about psychopathology, its nature, assessment, treatment, and cures. Second, improved resolution and data scalability can help monitor “within-person” change in ways existing measures cannot. In this regard, people can be tracked over time to best match them to interventions to minimize invasiveness, side-effects, and cost and to maximize their effectiveness (89, 90). Third, accounting for signal variation can help identify noise due to technical and recording issues, which can help efforts to optimize recording protocols. Knowing, for example, the level of unexplained variance in audio or video recordings across time/space can provide critical information for the recording equipment, software and conditions being used. Unexplained noise can even potentially be included as a measure of uncertainty when modeling. Finally, improved resolution can help facilitate integration across data streams such that complex systems can be modeled and understood. In the 21st century, technological and methodological advances hold the promise to revolutionize our understanding, assessment and treatment of psychopathology. However, to capitalize on these advances, we will need psychometrics that can help evaluate these technologically and methodologically sophisticated tools.

## Data Availability Statement

The data from this study will not be made publicly available because the research subjects did not consent to share their potentially identifying audio recordings or non-identifying data with anyone outside of our research team.

## Ethics Statement

The studies involving human participants were reviewed and approved by Louisiana State University IRB. The patients/participants provided their written informed consent to participate in this study.

## Author Contributions

AC, CC, and BE wrote the majority of the manuscript. RT, KM, ES, TL, PF, and TH contributed intellectual material to the project, helped interpret the extant literature, provided conceptual material, and assisted with the planning and presentation of the project. All authors approved the final manuscript.

## Conflict of Interest

The authors declare that the research was conducted in the absence of any commercial or financial relationships that could be construed as a potential conflict of interest.
