# Supplementary material for: Validating Biobehavioral Technologies for Use in Clinical Psychiatry
Source: Front Psychiatry. 2021 Jun 11;12:503323. doi: 10.3389/fpsyt.2021.503323 (PMC8225932; doi:10.3389/fpsyt.2021.503323)
Supplement: Supplementary file 1 [file Table_1.docx]

SUPPLEMENTAL MATERIALS

| Table S1: Coefficient values for logistic regression of acoustic features predicting momentary self-injurious thoughts/behaviors. | | | | |
| --- | --- | --- | --- | --- |
|  | |  |  |  |
|  | Estimate | Std. Error (robust) | z value | Pr(>\|z\|) |
| (Intercept) | -0.93 | 0.22 | -4.23 | 0.00 |
| Pause Mean | -0.23 | 0.27 | -0.85 | 0.40 |
| N Utterances | 0.18 | 0.20 | 0.89 | 0.37 |
| Pitch | 0.90 | 0.15 | 6.10 | 0.00 |
| Intonation | 0.53 | 0.13 | 4.08 | 0.00 |
| Jitter | -0.60 | 0.18 | -3.33 | 0.00 |
| Emphasis | -0.60 | 0.19 | -3.16 | 0.00 |
| Shimmer | 0.53 | 0.16 | 3.34 | 0.00 |
| F1 Variability | 0.42 | 0.19 | 2.25 | 0.02 |
| F2 Variability | -0.26 | 0.16 | -1.60 | 0.11 |
